# The combination of baseline neutrophil to lymphocyte ratio and dynamic changes during treatment can better predict the survival of osteosarcoma patients

**DOI:** 10.3389/fonc.2023.1235158

**Published:** 2023-11-14

**Authors:** Longqing Li, Ye Li, Minxun Lu, Yitian Wang, Zhuangzhuang Li, Xin Hu, Xuanhong He, Taojun Gong, Yi Luo, Yong Zhou, Li Min, Chongqi Tu

**Affiliations:** Department of Orthopedics, Orthopedics Research Institute, West China Hospital, Sichuan University, Chengdu, China

**Keywords:** neutrophils-to-lymphocytes ratio, osteosarcoma, survival, risk model, prognosis

## Abstract

**Background:**

Osteosarcoma is a primary malignant bone tumor with a high metastatic potential that accounts for a significant proportion of all bone tumors. The prognosis for patients with metastatic or recurrence disease remains poor. The neutrophil-to-lymphocyte ratio (NLR) has become a potential prognostic biomarker for cancer. Recent evidence suggests that the dynamic changes in neutrophil-to-lymphocyte ratio (NLR) during treatment may be more informative in predicting patient prognosis, but the value of dynamic NLR in osteosarcoma has not yet been determined.

**Methods:**

This retrospective study retrospectively analyzed the clinical information of 251 osteosarcoma patients diagnosed and treated in West China Hospital of Sichuan University, explored the impact of baseline NLR and changes in NLR during treatment on the prognosis of osteosarcoma patients, and further combined baseline NLR with Delta NLR to build an NLR staging system.

**Results:**

The results showed that both baseline NLR and delta NLR had some predictive ability for the prognosis of osteosarcoma patients (P = 6.90e-4, P = 0.022). Patients with high baseline NLR were more likely to have a decrease in delta NLR (P = 1.24e-10). The NLR stage had a better predictive ability than baseline NLR and delta NLR, and was an independent prognostic factor for overall survival in osteosarcoma patients HR: 2.456 (1.625-3.710) (P = 1.97e-05).

**Conclusion:**

NLR has value in continuous monitoring, and continuous monitoring of NLR can better predict the survival of osteosarcoma patients compared to baseline NLR.

## 1 Introduction

Osteosarcoma is a primary malignant bone tumor with a high metastatic potential that accounts for a significant proportion of all bone tumors (1, 2). Although chemotherapy has significantly improved the five-year survival rate of patients with non-metastatic osteosarcoma, the prognosis for patients with metastatic disease remains poor (3). Approximately 15-20% of affected patients already have metastases at presentation, and individuals with metastatic disease have low short- and long-term survival (4, 5). Furthermore, tumor recurrence and chemoresistance are also recognized as important prognostic factors (6). These clinical features highlight the need for improved diagnosis and treatment options for osteosarcoma (7). In recent years, advancements in imaging technology and molecular biology have allowed for more accurate diagnosis and better understanding of the molecular mechanisms underlying osteosarcoma (8, 9). Additionally, the use of targeted therapies, such as immune checkpoint inhibitors and kinase inhibitors, has shown promising results in preclinical studies and clinical trials (10–12). However, despite these advancements, there is still much to be done in terms of improving diagnosis and treatment options for osteosarcoma. For instance, the identification of biomarkers that can predict treatment response and patient outcome would greatly aid in tailoring treatment to individual patients (13–15).

With the continuous advancement of high-throughput technologies, recent research has made significant efforts to explore the pathogenic mechanisms of osteosarcoma and develop reliable therapeutic targets and robust prognostic markers (16, 17). The results of multiple Next-Generation Sequencing (NGS) and Genome-Wide Association Studies (GWAS) have deepened researchers’ understanding of the pathogenic mechanisms of osteosarcoma. For example, the frequent mutations in genes such as TP53 and PTEN, abnormal expression of MMP family genes, the role of the WNT/β-catenin pathway, the PI3K/AKT/mTOR signaling pathway, and the extracellular matrix remodeling pathway have all been highlighted as crucial factors in the occurrence and development of osteosarcoma, with potential as therapeutic targets (18, 19). Unfortunately, due to the additional costs associated with high-throughput technologies and differences in experimental protocols and analysis techniques across different laboratories, these findings have yet to be translated into clinical practice, especially concerning their utility as prognostic markers. In terms of prognostic biomarkers, recent research has confirmed the significant role of systemic inflammatory response in the occurrence and development of various tumors. Hematological markers of systemic inflammatory response, such as Neutrophil-to-Lymphocyte Ratio (NLR), Platelet-to-Lymphocyte Ratio (PLR), Lymphocyte-to-Monocyte Ratio (LMR), and Systemic Inflammatory Index (SII), have also gained widespread recognition for their prognostic value in predicting the outcomes of cancer patients (20–22). Importantly, these assessments are routinely obtained through standard clinical tests and often do not require additional testing costs. Among these, NLR research is the most extensive and has been incorporated into the latest guidelines for the diagnosis and treatment of urological system tumors (15, 23–26). Although most studies examining the role of NLR in cancer have focused on baseline values, recent evidence suggests that the dynamic changes in NLR during treatment may be more informative in predicting patient prognosis (27–29). Chronic inflammation and immune dysfunction are hallmarks of cancer, and NLR reflects the interaction between these two processes. The potential for dynamic changes in NLR to improve patient management has already been demonstrated in breast cancer and non-small cell lung cancer, but the value of dynamic NLR in osteosarcoma has not yet been determined (27, 30).

We hypothesize that dynamic NLR has potential prognostic value in osteosarcoma and consider NLR as a tumor biomarker. In this study, we retrospectively analyzed the NLR values and changes before and after neoadjuvant chemotherapy in osteosarcoma patients and preliminarily determined its prognostic value in osteosarcoma.

## 2 Patients and methods

### 2.1 Patients

After obtaining approval from the Medical Ethics Committee, we retrospectively analyzed the clinical data of osteosarcoma patients treated between January 2016 and January 2022 at the Musculoskeletal Tumor Center of West China Hospital. Inclusion criteria were patients with high-grade osteosarcoma confirmed by histopathology, complete hematological test results before neoadjuvant chemotherapy, and who received standard treatment at our hospital. Exclusion criteria were patients with low-grade osteosarcoma (intramedullary and bone surface) and periosteal osteosarcoma, those who had received neoadjuvant chemotherapy before their first visit, hematological diseases, other malignancies, and patients who did not receive standard treatment. A total of 251 patients were included in our study. Patients were followed up regularly until death or January 2023, with follow-up examinations conducted every 3 months within 1 year after surgery, every 4 months 1-2 years after surgery, every 5 months 2-3 years after surgery, every 6 months 3-5 years after surgery, and every year more than 5 years after surgery.

### 2.2 Data collection and processing

In order to meet clinical application requirements, neutrophil-to-lymphocyte ratio (NLR) was calculated based on the neutrophil and lymphocyte counts at the following time points and defined as baseline NLR and delta NLR. 1. Baseline NLR: calculated based on the first complete blood count before starting any treatment; 2. Delta NLR: calculated based on the last complete blood count before surgery after neoadjuvant chemotherapy (31). The result was obtained by subtracting the baseline NLR, and divided into two categories: increased or decreased. It is worth noting that we excluded the test results of using white blood cell-boosting agents, such as recombinant human granulocyte colony-stimulating factor, through the electronic medical record system of the patients. Therefore, some confounding factors may have been partially excluded.

We also retrospectively analyzed clinical information such as age, gender, tumor site, metastatic status and pathological fractures. It should be mentioned that according to previous research, patients with proximal fibular osteosarcoma may have a relatively poor prognosis. Therefore, this study singled out proximal fibular osteosarcoma and divided the tumor location into three parts: extremities, proximal fibula, and non-extremities. The tumor metastasis status is defined as whether the patient has confirmed metastasis at the initial visit.

### 2.3 Primary outcomes

The primary outcome is to investigate the correlation between NLR, delta-NLR and overall survival in osteosarcoma patients receiving standard treatment. The overall survival of osteosarcoma patients was defined as the interval from the start of any treatment to the patient’s death. Patients who were still alive at the time of the final follow-up were censored for the final analysis. The study also aims to explore the relationship and combination of NLR and delta-NLR with OS.

### 2.4 Statistical analysis

The statistical analysis was performed using R version 4.2.2 (R Foundation for Statistical Computing). The Kolmogorov-Smirnov test was used to assess the normality of continuous variables, and data were presented as mean, standard deviation, or proportion based on this. T-test or Mann-Whitney U test were used to evaluate differences between continuous variables depending on the results. The optimal cutoff value of NLR was calculated based on the receiver operating characteristic (ROC) curve. Establish NLR stage by combining NLR and delta NLR. Use time-dependent ROC curves to calculate the predictive ability of NLR stage, NLR, and delta NLR. The best predictor was used for further analysis. Kaplan-Meier plots and log-rank tests were used to compare survival rates. Multivariate Cox regression analysis was performed to identify independent prognostic factors for OS in osteosarcoma patients. Additionally, a nomogram was constructed to predict patient OS. A two-sided p-value of 0.05 or less was considered significant.

## 3 Results

### 3.1 Baseline characteristics

Demographic and disease characteristics of the 251 osteosarcoma patients who met the inclusion and exclusion criteria are shown in **Table 1**. The mean age was 21 years (range, 5-67 years) with 145 (57.8%) male and 106 (42.2%) female patients. Tumors were located in the extremities in 239 (95.2%) patients, including 12 (4.8%) patients with tumors in the proximal fibula, while only 12 (4.8%) patients had tumors located in non-extremity sites. Thirty (12%) patients had pathological fractures at the time of initial diagnosis, and 42 (16.7%) patients were confirmed to have metastasis at the time of initial diagnosis.

**Table 1:** Baseline of clinical characteristics of osteosarcoma patient in the study.

Characteristic	N (%), N = 251
Age	
Mean (Range)	21.3(5-67)
Gender	
Male	145 (57.8%)
Female	106 (42.2%)
Metastasis.status	
No	209(83.3%)
Yes	42 (16.7%)
Tumor.site	
Extremities	227(90.4%)
Proximal fibula	12(4.8%)
Non-extremities	12(4.8%)
Pathological.fracture	
No	221(88%)
Yes	30(12%)

### 3.2 Predictive value of baseline NLR and delta NLR on overall survival in osteosarcoma patients

**Figure 1:**
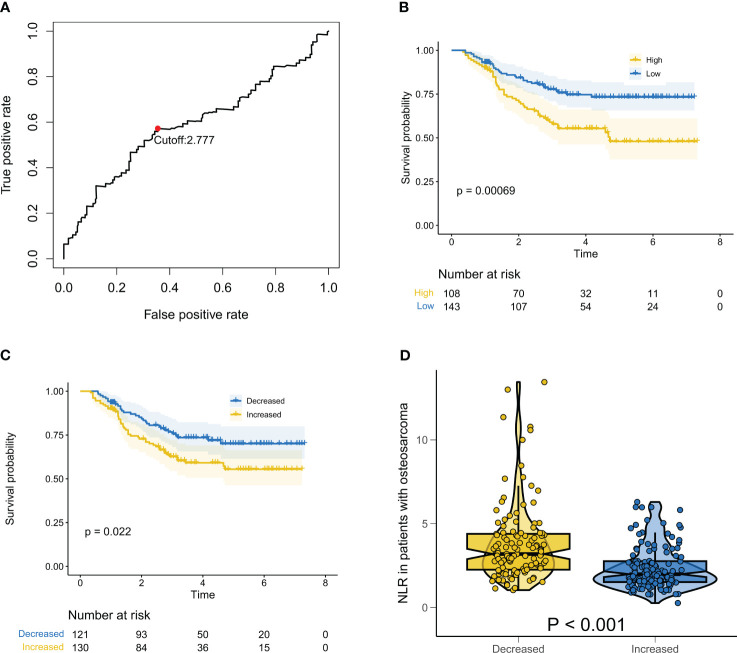
Relationship between baseline NLR and Delta NLR and prognosis of patients with osteosarcoma. **(A)** ROC results showing the optimal cutoff value for baseline NLR; **(B)** Kaplan-Meier survival curve for overall survival in patients with osteosarcoma at different baselines NLR; **(C)** Kaplan-Meier survival curve for overall survival in patients with osteosarcoma at different delta NLR; **(D)** Box violin plot showing the difference of baseline NLR of patients in different Delta NLR groups.

As mentioned earlier, a cutoff value of 2.77 was defined for baseline NLR based on the ROC results (**Figure 1A**). Compared to patients with NLR values lower than or equal to 2.77, those with baseline NLR values greater than 2.77 were significantly associated with a shorter median OS (**Figure 1B**, P = 6.90e-4). As expected, delta NLR also had some predictive value, with an increase in delta NLR being associated with a lower median OS than a decrease in delta NLR (**Figure 1C**, P = 0.022). We then compared the baseline NLR values of patients in different delta NLR groups. As shown in **Figure 1D**, the baseline NLR values of patients with an increase in delta NLR were significantly lower than those with a decrease in delta NLR (P = 1.24e-10).

### 3.3 Construct NLR stage by combining baseline NLR and delta NLR

**Figure 2:**
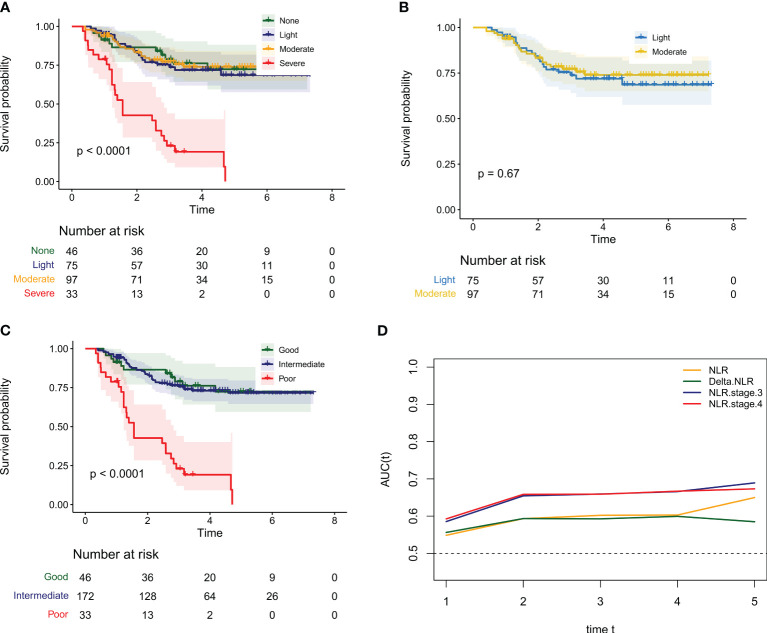
The NLR stage combining the baseline NLR and Delta NLR can more accurately identify the prognosis of osteosarcoma patients. **(A)** Kaplan-Meier survival curves for the overall survival of osteosarcoma patients in different groups when the NLR stage is divided into four groups; **(B)** Kaplan Meier survival curves for the overall survival of osteosarcoma patients in the Light and Moderate groups when the NLR stage is divided into four groups; **(C)** Kaplan-Meier survival curves for the overall survival of osteosarcoma patients in different groups when the NLR stage is divided into three groups; **(D)** The time dependent ROC curve of AUC value change of baseline NLR, Delta NLR and NLR stage at different time points.

We hypothesized that combining baseline NLR and delta NLR may better predict the survival of osteosarcoma patients. Firstly, patients were classified into four groups based on their baseline NLR and delta NLR: None (low baseline and decrease in delta NLR), Light (high baseline and decrease in delta NLR), Moderate (low baseline and increase in delta NLR), and Severe (high baseline and increase in delta NLR). As we hypothesized, the OS median of the Severe group was significantly lower than the other three groups (**Figure 2A**, P < 0.0001). Unexpectedly, there was no significant difference in prognosis between the Light and Moderate groups (**Figure 2B**, P = 0.670). Therefore, we attempted to classify patients into three groups: Good, with low baseline and decrease in delta NLR; Poor, with high baseline and increase in delta NLR; Intermediate, for the remaining two groups of patients. As expected, classifying patients into three groups had similar predictive ability as the four groups (**Figure 2C**, P < 0.0001). Subsequently, we used time-dependent ROC curves to evaluate the predictive ability of the three groups, four groups, baseline NLR, and delta NLR for overall survival of osteosarcoma patients at different time points. As shown in **Figure 2D**, the predictive ability of the three groups and four groups was significantly higher than baseline NLR and delta NLR at all time points. However, there was no significant difference between the three groups and four groups. Considering that there was no significant difference in overall survival between the Light and Moderate groups, we finally selected the three-group NLR stage for further analysis. Our results indicate that the positive predictive value (PPV), negative predictive value (NPV), sensitivity (SEN), and specificity (SPE) for NLR grading are as follows: PPV: 0.818 (0.687-0.950), NPV: 0.757 (0.700-0.814), SEN: 0.338 (0.234-0.441), SPE: 0.965 (0.937-0.992).

### 3.4 Predictive ability of NLR stage and clinical characteristics

**Figure 3:**
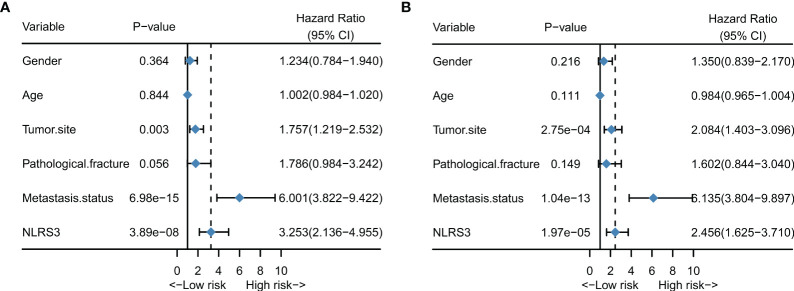
NLR stage is an independent prognostic factor for patients with osteosarcoma. **(A)** Forest plot showing the results of univariate cox regression analysis for patients with osteosarcoma; **(B)** Forest plot showing the results of multivariate cox regression analysis for patients with osteosarcoma.

We used Cox regression models to investigate the impact of NLR stage and clinical characteristics on overall survival of osteosarcoma patients, and conducted multivariate analysis to explore independent predictors of overall survival in osteosarcoma patients. The results of univariate analysis showed that NLR stage, metastatic status, and tumor site were significantly associated with overall survival in osteosarcoma patients. The hazard ratios for these three variables were: NLR stage HR: 3.253 (2.136-4.955) (P = 3.89e-08); metastatic status HR: 6.001 (3.822-9.422) (P = 6.98e-15); tumor site HR: 1.757 (1.219-2.532) (P = 0.003) (**Figure 3A**).

After adjusting for all variables in the multivariate analysis, NLR stage, tumor site, and metastatic status were identified as independent prognostic factors associated with survival in osteosarcoma patients. The hazard ratios were as follows: NLR stage HR: 2.456 (1.625-3.710) (P = 1.97e-05); metastatic status HR: 6.135 (3.804-9.897) (P = 1.04e-13); tumor site HR: 2.084(1.403-3.096) (P = 2.75e-04) (**Figure 3B**).

### 3.5 Construct nomogram for predicting overall survival of osteosarcoma patients

**Figure 4:**
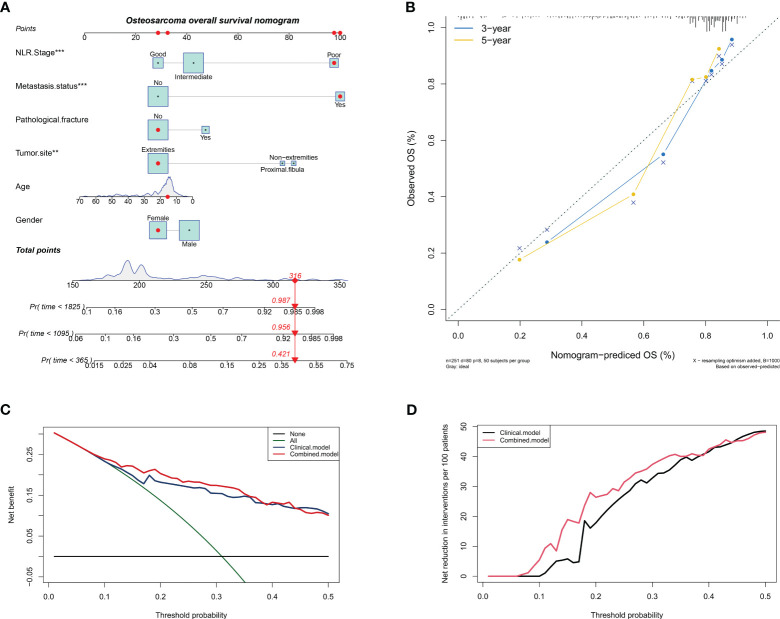
Constructing a column chart that can predict overall survival of patients with osteosarcoma. **(A)** A nomogram for predicting overall survival of osteosarcoma patients by introducing NLR stage; **(B)** Calibration chart to verify the accuracy of the nomogram; **(C)** Net benefit curve of nomogram; **(D)** Net reduction curve of nomogram.

Nomogram have become increasingly popular in clinical research because they can be used to individualize risk prediction and inform treatment decisions. Based on the above results, we combined NLR stage with clinical characteristics to construct a nomogram that can predict the overall survival of osteosarcoma patients. As shown in **Figure 4A**, the nomogram assigns a score to each variable according to its importance, where NLR stage and tumor metastasis status have a similar score range, once again indicating the good predictive ability of NLR stage. The C-index of the nomogram is 0.797, and together with the results of the calibration curve, it indicates that the nomogram has good predictive accuracy in predicting the overall survival of osteosarcoma patients (**Figure 4B**). Finally, the results of clinical decision analysis showed that the nomogram with NLR stage introduced brought clinical net benefits compared to the model with clinical characteristics alone (**Figures 4C, D**).

## 4 Discussion

To our knowledge, our study is the first to investigate the prognostic value of NLR changes during treatment in patients with osteosarcoma. Previous studies have mainly focused on the baseline NLR value as a prognostic factor in osteosarcoma (32–34). In this study, by observing the NLR change trend after neoadjuvant chemotherapy, as we hypothesized, the increase of NLR during treatment was associated with poor prognosis in osteosarcoma patients. This result suggests that NLR may have the potential to serve as a biomarker similar to tumor markers in osteosarcoma patients, with value for continuous monitoring. More importantly, this study is the first to combine baseline NLR with delta NLR to construct an NLR stage system. The NLR grading system has significantly better predictive ability for overall survival in osteosarcoma patients than baseline NLR and delta NLR alone, further demonstrating the potential of NLR as a biomarker with continuous monitoring value.

As expected, both baseline NLR and delta NLR showed prognostic value. However, contrary to previous studies, delta NLR did not demonstrate superior potential to baseline NLR (27). We believe the main reason is that baseline NLR was significantly higher in patients whose delta NLR was decreased, compared to those whose delta NLR increased. Standard treatment for osteosarcoma patients includes 2-3 cycles of neoadjuvant chemotherapy prior to surgery, which inevitably affects the patient’s immune cell population (35). However, previous studies have shown that the degree of chemotherapy’s impact on different immune cells is not consistent, with a greater reduction observed in neutrophils compared to lymphocytes (29). This leads to a decrease in delta NLR in most patients with high baseline NLR, and the poor prognosis of patients with high baseline NLR is already widely recognized. This contradiction to some extent reduces the predictive ability of delta NLR and highlights its limitations when used alone to predict patient prognosis.

Due to the complexity of tumor occurrence and progression, it is difficult for imaging or laboratory tests to accurately predict the prognosis of cancer patients at a single time point (36, 37). Previous studies and our research results have preliminarily confirmed the value of NLR in dynamic monitoring (38, 39). To fully utilize the predictive ability of NLR, our study combined baseline NLR and delta NLR to construct NLR stage. It is encouraging that NLR stage has significantly better prognostic value than baseline NLR and delta NLR. According to our research results, some patients with poor prognosis due to high baseline NLR may be “saved,” which is consistent with previous research results (31). The prognosis of patients in the NLR stage with high baseline NLR and decreased delta NLR is not significantly different from that of patients with low baseline NLR, suggesting that normalization of NLR during treatment may have the potential to reflect patient response to treatment. Patients with high baseline NLR combined with high delta NLR have a significantly worse prognosis than other groups, indicating that NLR grading can more accurately identify truly high-risk patients.

.Previous studies have shown that NLR is correlated with the prognosis of cancer patients mainly because high NLR may reflect a pro-inflammatory state that promotes angiogenesis, inhibits cell apoptosis, DNA damage and higher levels of circulating cytoplasmic division (40, 41). Recently, studies have also provided reliable biological evidence for NLR in predicting the prognosis of cancer patients. High neutrophil counts are associated with the release of pro-tumor substances such as reactive oxygen species, arginase, inflammatory cytokines, tumor or vascular growth factors, and metalloproteinases, while low lymphocyte counts are associated with impaired anti-tumor response, CD8+ T cell cytotoxicity, and CD4+ helper T cell function (42–44). Therefore, NLR is an expression of the global balance between pro-tumor inflammation and anti-tumor immunity.

**Figure 5:**
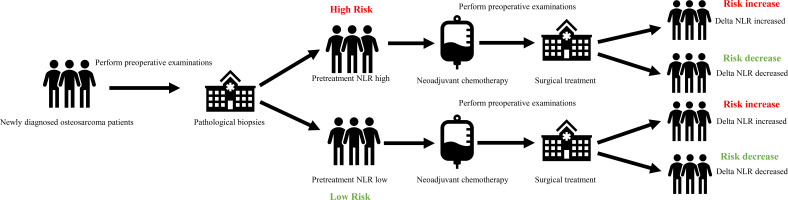
Brief process of hematological testing and calculation.

It is undeniable that this study has some limitations. Firstly, as a retrospective study, selection bias may exist in our study. This is also a common limitation in the study of the value of hematological inflammation indicators in skeletal muscle tumors (**Table 2**). However, we believe that this does not negate the value of some aspects of our study. **Figure 5** displays the collection time points for NLR and Delta NLR in our retrospective study. It can be observed that the collection of these markers is straightforward, easily replicable, and stems from routine preoperative testing without additional testing costs. What’s more, compared to new testing methods like high-throughput sequencing, the results of hematological tests can readily be applied in clinical settings without being affected by batch effects across different platforms. Therefore, the primary importance of this study is to provide new evidence for the continued monitoring value of NLR as a biomarker in osteosarcoma. Secondly, the cutoff value of NLR was calculated based on our cohort and may not be fully applicable to other cohorts, although our results indicate that NLR as a continuous variable also has similar predictive value. Finally, the timing of delta-NLR was set preoperatively and may ignore some time points during neoadjuvant chemotherapy. However, we believe that as the first study to report the impact of NLR changes during treatment on the prognosis of osteosarcoma patients, our results still have important clinical significance. Further research is needed to validate and strengthen our conclusions. For instance, prospective studies on the value of pretreatment NLR, delta NLR, and NLR grading in osteosarcoma, or studies correlating the effectiveness of different treatment regimens in osteosarcoma patients, as well as the value of dynamic changes in inflammation markers such as PLR and LMR in osteosarcoma.

**Table 2:** An overview of hematological inflammatory biomarkers in osteosarcoma and soft tissue sarcoma research.

Marker	Diagnosis	Formula	Test time	Research type
NLR	OS/STS	Neutrophils/Lymphocytes	Pretreatment	Retrospective
PLR	OS/STS	Platelets/Lymphocytes	Pretreatment	Retrospective
LMR	OS/STS	Lymphocytes/Monocytes	Pretreatment	Retrospective
CAR	OS/STS	C-reactive protein/Albumin Ratio	Pretreatment	Retrospective
dNLR	OS/STS	Neutrophil/(White blood cell count - neutrophil)	Pretreatment	Retrospective
SSI	OS/STS	Platelets * Neutrophils/Lymphocytes	Pretreatment	Retrospective

OS, osteosarcoma; STS, Soft tissue sarcoma.

## 5 Conclusion

In conclusion, our results indicate that the increase of NLR during the treatment process is associated with poor prognosis in osteosarcoma patients. The combination of baseline NLR and delta NLR into an NLR stage can more accurately predict overall survival in osteosarcoma patients.

## Data availability statement

The original contributions presented in the study are included in the article/supplementary material. Further inquiries can be directed to the corresponding authors.

## Ethics statement

The studies involving humans were approved by Medical Ethics Committee of the West China hospital. The studies were conducted in accordance with the local legislation and institutional requirements. Written informed consent for participation in this study was provided by the participants’ legal guardians/next of kin.

## Author contributions

LL and YLi collected and analyzed the data and wrote the paper. ML and YW assisted in collecting the data and participated in the writing. ZL, XH, XHH, TG, YLu, and YZ assisted in the design of this study. LM and CT are responsible for all the integrity of data and the accuracy of data analysis. All authors have thoroughly revised the manuscript.
